# Changes in the proportion of anemia among young women after the Great East Japan Earthquake: the Fukushima health management survey

**DOI:** 10.1038/s41598-022-14992-3

**Published:** 2022-06-25

**Authors:** Kana Yamamoto, Morihito Takita, Masahiro Kami, Yoshinobu Takemoto, Tetsuya Ohira, Masaharu Maeda, Seiji Yasumura, Akira Sakai, Mitsuaki Hosoya, Kanako Okazaki, Hirooki Yabe, Toshio Kitamura, Masaharu Tsubokura, Michio Shimabukuro, Hitoshi Ohto, Kenji Kamiya

**Affiliations:** 1 Department of Internal Medicine, Graduate School of Medicine, The University of Tokyo, Minato, Tokyo 108-0071 Japan; 2 Department of Radiation Health Management, Fukushima Medical University, Fukushima, Fukushima 960-1295 Japan; 3 Department of Internal Medicine, Navitas Clinic Tachikawa, Tachikawa, Tokyo 190-0023 Japan; 4 Department of Internal Medicine, Medical Governance Research Institute, Minato, Tokyo 108-0074 Japan; 5 Department of Internal Medicine, Yoshinobu Clinic, Kagoshima, 890-0063 Japan; 6 Radiation Medical Science Center for the Fukushima Health Management Survey, Fukushima Medical University, Fukushima, Fukushima 960-1295 Japan; 7 Department of Epidemiology, Fukushima Medical University, Fukushima, Fukushima 960-1295 Japan; 8 Department of Disaster Psychology, Fukushima Medical University, Fukushima, Fukushima 960-1295 Japan; 9 Department of Public Health, Fukushima Medical University, Fukushima, Fukushima 960-1295 Japan; 10 Department of Radiation Life Sciences, Fukushima Medical University, Fukushima, Fukushima 960-1295 Japan; 11 Department of Pediatrics, Fukushima Medical University, Fukushima, Fukushima 960-1295 Japan; 12 Department of Neuropsychiatry, Fukushima Medical University, Fukushima, Fukushima 960-1295 Japan; 13 Division of Cellular Therapy, The Institute of Medical Science, The University of Tokyo, Minato, Tokyo 108-0071 Japan; 14 Department of Diabetes, Endocrinology and Metabolism School of Medicine, Fukushima Medical University, Fukushima, Fukushima 960-1295 Japan; 15 Research Institute for Radiation Biology and Medicine, Hiroshima University, Hiroshima, 734-8553 Japan

**Keywords:** Natural hazards, Health care, Medical research

## Abstract

This study aimed to evaluate the sequential changes in the proportion of anemia among young women over eight years after the Great East Japan Earthquake in 2011 using a prospective study of the Fukushima Health Management Survey. This study focused on the women aged between 20 and 44 who lived in the evacuation area of the nuclear power plant accident. The yearly age-adjusted proportion of anemia was accessed with data between July 2011 and March 2019. A total of 9,198 women participated in the health checkup in 2011, albeit the participation was decreased to 1,241 in 2018. The age-adjusted proportion of anemia was 16.7% in 2012 and then declined after 2013 (*p* with Cochran-Armitage trend test = 0.03). The multivariate regression analysis identified < 23 kg/m^2^ of body mass index (BMI), no history of smoking, and no habitual alcohol use as independent baseline characteristics predictive of temporality anemic condition after the disaster (Adjusted odds ratios [95% confidence interval]; 1.98 [1.43–2.74], 1.85 [1.21–2.83], and 1.42 [1.07–1.90], respectively). Thus, women with low BMI and healthier habits might risk temporarily anemic status after the disaster. Our findings signal the importance of preventing anemia in young women after the disaster.

## Introduction

Anemia is a medical condition commonly seen in clinical practice, where the number of red blood cells and, consequently, their oxygen-carrying capacity is insufficient to meet physiological needs^1^. The most common cause of anemia is iron deficiency^2^. The bone marrow damage, blood loss, inflammation, metabolic abnormalities, and hemolysis also develop anemia. In a Japanese study, the prevalence of anemia among women aged 20 − 49 years was 17.1%, and 0.2% of them had severe anemia, which is less than 8.0 g/dL of hemoglobin level^3^. Of note, the prevalence of anemia in young women has been increasing in developed countries^4^. There are two major causes of anemia in young women: excessive blood loss through menstruation and insufficient red cell production due to inadequate dietary intake of iron^2^. The anemia prevalence trended upward in women with a lower body mass index (BMI)^5^. Our recent report showed a consistent finding where the prevalence of anemia reached 18.2% with BMI < 18.5 kg/m^2^ and declined to 9.9% with BMI ≥ 30.0 kg/m^2^ among young women in Shanghai^6^. The average BMI of Japanese women has decreased consistently since 1945^7^. Thus, anemia has been a serious and unsolved public health concern.

Natural disasters directly impact injuries, exposure to harmful substances, diseases, and mental health. Additionally, they have significant impacts on human health by causing shortages or lack of necessities and services, including clean water, food, communication, and lack of medical resources^8^. Natural disasters can influence anemia. For example, the prevalence of anemia increased significantly among pregnant women in the areas affected by the 1997 Red River flood in North Dakota^9^. Likewise, one year after the 2008 Wenchuan Earthquake, the prevalence of anemia among child-bearing women was 28.8%^10^.

Additionally, the micronutrient status of Vitamin B12, Vitamin A, and zinc among women of reproductive age was poor in disaster areas^10^. Drought in Lake Urmia has severely affected hypertension and anemia in the local population^11^. Women exposed to the Chornobyl accident in 1986 were more likely to develop anemia after delivery^12^. However, the long-term changes in anemia status in the evacuees after the disaster are difficult to demonstrate since they move from their original residence to unfamiliar locations, making it difficult to track their health status^13^.

The Great East Japan Earthquake, followed by huge tsunamis, occurred on March 11, 2011. The subsequent nuclear accident at the Fukushima Daiichi Nuclear Power Plant (FDNPP) resulted in the widespread release of radioactive materials^14^. In addition to health risks such as physical injuries, infection, and mental illness due to natural disasters and the evacuation^15,16^, the residents nearby FDNPP were particularly at risk of radiation exposure which may cause the acute and late phase of radiation sickness^17,18^. In response to this situation, the Prefecture Government launched the Fukushima Health Management Survey (FHMS) to investigate the health status of residents and utilize the data obtained for health promotion in June 2011, with a target population of approximately 2.05 million^19^. The FHMS consists of five components: a primary survey, a thyroid ultrasound examination, a comprehensive health check, a mental health and lifestyle assessment, and a pregnancy and birth survey.

The FHMS studies have revealed the changes in the health status of residents forced to evacuate for extended periods. Due to the evacuation and subsequent lifestyle changes in the residents, an increased risk of lifestyle-related diseases, including obesity, dyslipidemia, and diabetes, was observed^20–22^. The effects of the disaster also impacted dietary habits as the intake of meat and fish among young people decreased after the disaster^23^. Concerns over potential contamination of agricultural products produced in the Fukushima Prefecture among pregnant women in Minamisoma nearby FDNPP led to a reduction in consumption of rice, vegetables, and fruits, following the disaster^24^. There are no reports of anemia in the evacuees after the Great East Japan Earthquake, even though anemia is a common disease. Given the previous findings on changes in metabolic and nutritional changes in the evacuees, we hypothesized that a lowering of the anemia proportion would be observed over time after the disaster, which means the presence of the time-dependent trend in the anemia proportion. This study investigated the proportions of anemia and the characteristics of evacuees with anemia among young women between July 2011 and March 2019 using the FHMS data.

## Materials and methods

### Study participants

Study participants included women between the ages of 20 and 44 who underwent a comprehensive health check in the FHMS between July 2011 and March 2019, a period of 4 to 96 months after the disaster. We focused on the survey participants who lived in the evacuation area of the Fukushima Daiichi Nuclear Power Plant accident before the disaster. The subjects given written consent for the study were included.

### Comprehensive health check

The comprehensive health check was conducted as a part of the FHMS, which was launched in July 2011 after the disaster^19^. This large-scale cohort study attempts to identify the effects of extended evacuation by reviewing health information on the evacuees and assessing the proportion of various diseases. The target population included residents of all age groups living in the evacuation zones between March 11, 2011, and April 1, 2012, designated by the government^25^. This included all of Tamura City, Minamisoma City, Kawamata Town, Hirono Town, Naraha Town, Tomioka Town, Kawauchi Village, Okuma Town, Futaba Town, Namie Town, Katsurao Village, Iitate Village, and a part of Date City. The participation rate of the comprehensive health check for all age groups ranged between 20.2% in 2018 and 35.4% in 2011 (S1 Table)^26^.

### Variables and definitions

The data obtained from the health checkup included basic information, such as residence at the time of the disaster, date of assessment, medical history, and lifestyle habits, such as drinking and smoking, height, weight, BMI and blood pressure, and blood tests measuring red cell count, hematocrit, and hemoglobin level. The hemoglobin levels were utilized to calculate the annual proportion of anemia among the study subjects, which is the primary outcome of this study. The baseline characteristics of age, BMI, blood pressure, disease history, history of smoking, and drinking habit in 2011 were treated as the candidates for exploratory variables associated with anemic status after the disaster. Little has been reported on characteristics associated with anemia in women of reproductive age after a disaster, although an international cross-sectional study demonstrated that age, BMI, and socioeconomic status could be associated with anemia in women of reproductive ages^27^. Smoking and drinking habits may be determinants of anemia in young women^28^.

Anemia was defined when hemoglobin levels were lower than 12.0 g/dL in women, following the World Health Organization (WHO) criteria^29^. Mean corpuscular volume (MCV), mean corpuscular hemoglobin (MCH), and mean corpuscular hemoglobin concentration (MCHC) were calculated based on complete blood cell counts^30^. We defined a drinking habit as when the study participants drank alcohol occasionally or every day. Relocation history was identified based on reported residences in 2011 before the disaster, compared to 2011 or 2012 after the disaster.

### Statistical analysis

Participant characteristics were summarized with descriptive statistics. We assessed the proportion of anemia in the subjects using the FHMS comprehensive health check. The proportion of anemia was calculated for each year of the study. Then, the proportion estimates between 2012 and 2018 were adjusted based on the 2011 age distribution. The adjusted annual proportion of anemia was plotted with the survey year. The linear regression was employed to plot the line of best fit. The trend of annual anemia proportion was assessed with the Cochran-Armitage test. Univariate analyses were performed to identify characteristics associated with the temporary anemia after the disaster using one-way analysis of variance (ANOVA) followed by a post hoc comparison using Tukey correction for continuous variables or Fisher’s exact test corrected by the Holm method for categorical variables. The multivariate logistic regression model employed a stepwise backward selection for variables in the univariate assessments that exhibited significant variation. Statistical significance was considered when the two-sided *p*-value was less than 0.05. All statistical analyses were performed with SPSS version 28 (IBM, Armonk, NY) except for Fisher’s exact test and Cochran-Armitage test, which used R (version 4.0.5) contained within RVAideMemoire (version 0.9–79) and DescTools (Version 0.99.44)^31^.

### Ethics approval

As part of their original participation in the FHMS comprehensive health program, the study participants provided written informed consent for exploratory analyses at the time of enrollment. We analyzed the FHMS data after approval from the Ethics Committee of Radiation Medical Science Center for the Fukushima health management (Fukushima, Japan; approval number: 2020239 for analysis of Mental Health and Lifestyle Survey, 1319 for Comprehensive Health Checkup, and 29,064 for analysis linking between Mental Health and Lifestyle Survey and Comprehensive Health Checkup in FHMS). This study was performed in accordance with the Helsinki Declaration and the Ethical Guidelines for Medical and Health Research Involving Human Subjects established by the Japanese Ministry of Health, Labour and Welfare.

## Results

### Participant Characteristics in 2011 after the disaster

A total of 9,198 women aged 20 to 44 years participated in the comprehensive health check with a complete blood count test between July 2011 and March 2012. We estimated the corresponding target population for this study as 25,058 women, which accounts for 36.7% of the participation rate (S2 Table). S1 Figure shows the changes in the number of participants. The participation of subjects decreased to 1,241 women in 2018, which is 13.5% of the first survey in 2011. We compared the baseline characteristics in 2011, classified by participation status after 2012 (S3 Table). The subjects with no participation after 2012 showed significantly smaller MCV compared to those who participated at least once or more (87.9 fL and 88.2 fL of the average, *p* = 0.019).

The study participant characteristics in 2011 are presented in Table 1 (S4 Table for missing data). The mean age (*standard deviation*) was 33.7 [6.7] years for women. The ratio of participants in the 35–39 age group was the highest compared to other age groups(25.4% in women). Mean BMI was normal weight (22.2 [4.2] for women)^32^. The smoking history and alcohol habit percentages were low among women (20.0% and 9.1%, respectively).

**Table 1:** Characteristics of women participants in the 2011 comprehensive health check.

	Female
	(*n* = 9,198)
Age-years	33.7 (*6.7*)
**Age group-*n* (*%*)**	
20–24 years	1,097 (*11.9*)
25–29 years	1,538 (*16.7*)
30–34 years	2,045 (*22.2*)
35–39 years	2,333 (*25.4*)
40–44 years	2,185 (*23.8*)
Body weight (kg)	55.6 (*10.9*)
Body mass index (kg/m^2^)	22.2 (4.2)
**Medical history-*n* (*%*)**	
Current hypertension	145 (*1.6*)
Cerebrovascular disease	20 (*0.2*)
Heart disease	96 (*1.1*)
Current diabetes treatment	55 (*0.6*)
Current treatment of dyslipidemia	59 (*0.7*)
Kidney disease	170 (*1.9*)
History of smoking-*n* (*%*)	1,797 (*20.0*)
Drinking habit of alcohol-*n* (*%*)	814 (*9.1*)
**Hematological characteristics**	
History diagnosed as anemia-*n* (*%*)	1,610 (*18.0*)
**Peripheral blood counts**	
Red blood cells (10^6^/μL)	4.54 (*0.33*)
Hemoglobin (g/dL)	13.1 (*1.4*)
Hematocrit (%)	39.9 (*3.3*)
**Red cell indices**	
MCV (fl)	88.1 (*6.3*)
MCV < 80-*n* (*%*)	872 (*9.5*)
MCV > 100-*n* (*%*)	79 (*0.9*)
MCH	28.9 (*2.7*)
MCHC	32.8 (*1.3*)

The average (standard deviation) or numbers (percentages) are shown. Abbreviations: mean corpuscular volume; MCV, mean corpuscular hemoglobin; MCH, and mean corpuscular hemoglobin concentration; MCHC.

### Proportion of anemia

**Figure 1:**
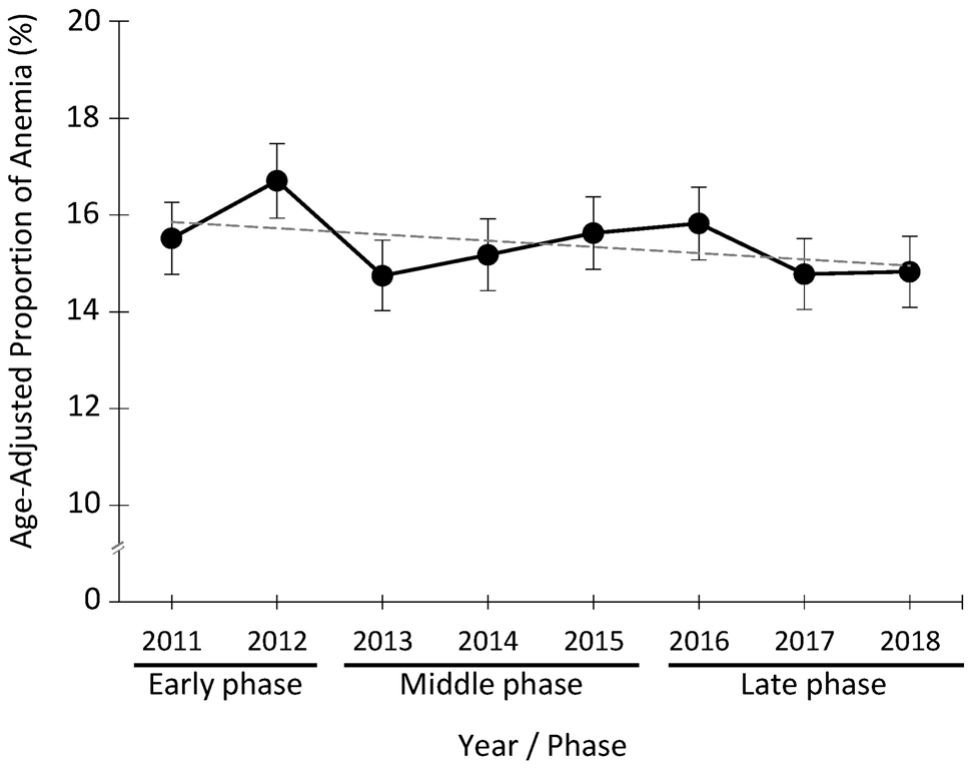
The proportion of anemia among women in the evacuation area after the Fukushima Daiichi Nuclear Power Plant accident. Percent change with 95% confidence intervals in the age-adjusted proportion of anemia after the Fukushima Daiichi nuclear disaster was shown among women who received a health check in 2011. The proportion estimates between 2012 and 2018 were adjusted based on the 2011 age distribution. Cochran-Armitage trend test output 0.03 of the *p*-value. The dashed line indicates linear regression, which showed a declining trend (slope =  − 1.3 × 10^−3^).

Figure 1 displays the yearly proportion of anemia in women from 2011 to 2018, where the yearly proportion was adjusted with the age distribution in 2011. Overall, the highest anemia proportion among the study subjects (16.7%) was observed in 2012, and the proportion gradually declined during the study period (Cochran-Armitage test for trend, two-sided *p* = 0.03 and the slope of linear regression =  − 1.3 × 10^−3^). S2 Figure shows the same data broken down by age groups.

**Figure 2:**
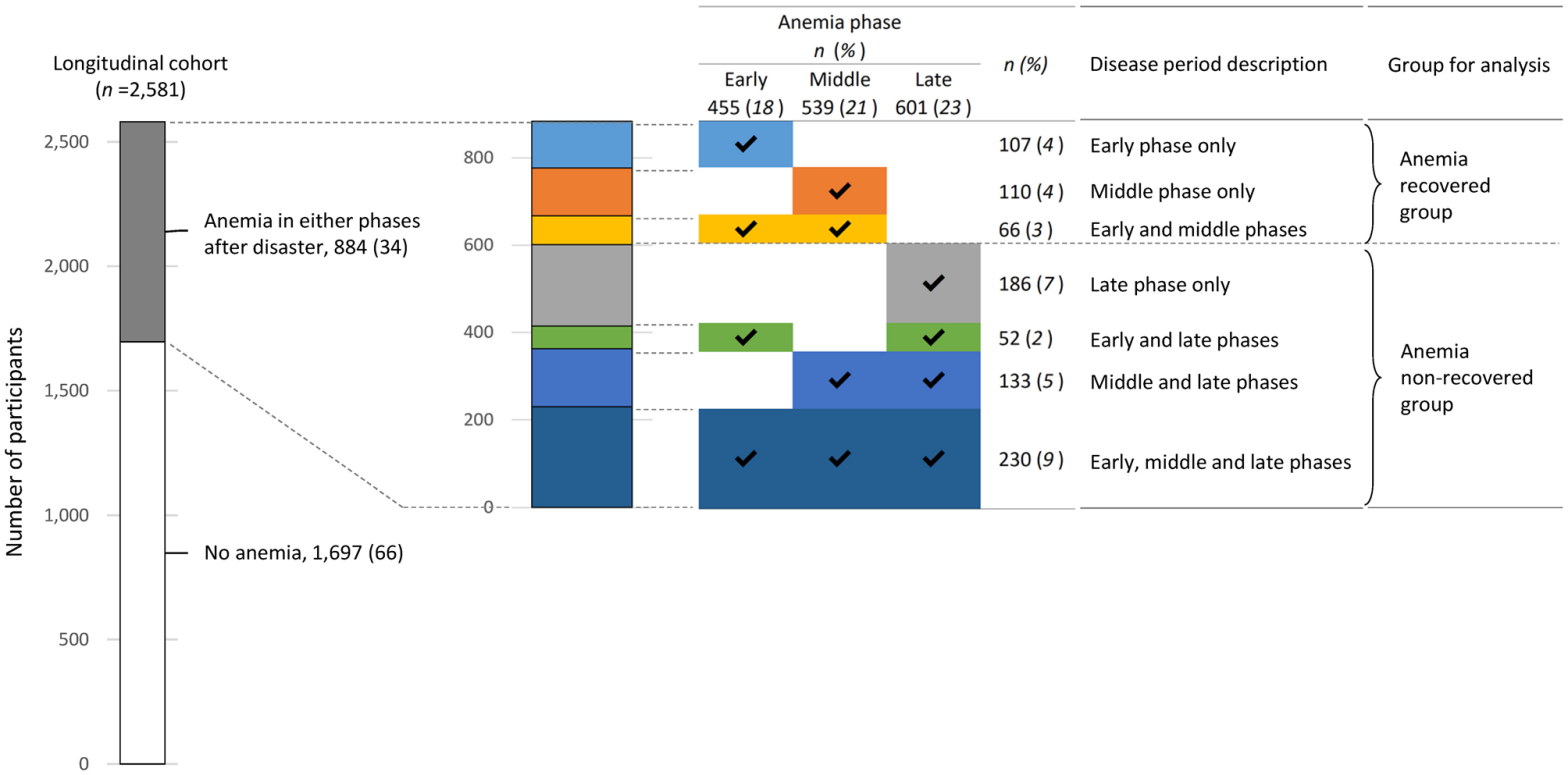
Classification of women participants by anemia phases post-disaster. The number and percentage of women participants classified by anemia phases after the disaster (*n* = 2,581 of the longitudinal cohort in total).

We focused on the patient characteristics in women associated with temporary anemia after the disaster. We defined the three phases post-quake: 2011 to 2012, 2013 to 2016, and 2017 to 2018, representing early, middle and late phases, respectively (Fig. 1). This classification of phases post-disaster was based on changes in the anemia proportion, modification of evacuation areas, and conceptional framework in the previous studies^33,34^. These three phases were further categorized into seven combinations of the disease period of anemia (Fig. 2). We selected women who underwent at least the health check for every phase in the longitudinal analysis (*n* = 2,581; Fig. 2) and compared the participant baseline characteristics between the seven combinations of disease periods (S5 Table). We then grouped the participants into the following three groups; those with no detection of anemia in the study period (‘no anemia’ group), anemia found in either early or middle phases but not in the late phase (‘anemia recovered’ group) and anemia seen in the late phase (‘anemia non-recovered’ group) (Fig. 2).

### Baseline characteristics in women associated with temporal anemia after the disaster

We compared the baseline characteristics of women among the no anemia, anemia recovered, and anemia-non recovered groups (Table 2). The anemia recovered group, which indicates those with temporal anemia after the disaster, exhibited significantly younger age at baseline when compared to the anemia non-recovered group (31.0 and 32.0 years of age, respectively; *p* = 0.017). The recovered group exhibited significantly lower body weight, BMI, and waist circumstance than the no anemia group (*p* = 0.006, 0.001, and 0.018, respectively). The lowest average systolic and diastolic blood pressures were observed in the recovered group (110 and 66 mmHg, respectively), showing statistical significance compared to the non-recovered group (*p* = 0.006 and 0.027).

**Table 2:** Characteristics of women participants with anemia classified by periods of occurrence.

Variables	Group			*p*-value	
	No anemia (A)	Anemia recovered (B)	Anemia	Overall	Post-hoc comparison
	(*n* = 1,697)	(*n* = 283)	non-recovered (C)		
			(*n* = 601)		
Age at disaster in 2011	30.7 (*5.0*)	31.0 (5.2)	32.0 (4.9)	< 0.001	< 0.001 for A *vs.* C, and 0.017 for B *vs.* C
Body weight (kg)	55.5 (*11.5*)	53.3 (8.5)	54.9 (11.0)	0.008	0.006 for A *vs.* B
Body mass index (kg/m^2^)	22.2 (*4.4*)	21.2 (3.3)	22.0 (4.2)	0.002	0.001 for A *vs.* B, and 0.033 for B *vs.* C
Waist (cm)	77.1 (10.4)	74.0 (8.9)	76.2 (9.8)	0.023	0.018 for A *vs.* B
Systolic blood pressure (mmHg)	111 (12)	110 (12)	113 (13)	0.002	0.007 for A *vs.* C, and 0.006 for B *vs.* C
Diastolic blood pressure (mmHg)	68 (10)	66 (10)	68 (10)	0.03	0.047 for A *vs.* B, and 0.027 for B *vs.* C
**Medical history-*n* (*%*)**					
Medication for hypertension	15 (*0.9*)	3 (*1.1*)	9 (*1.5*)	0.454	
Cerebrovascular disease	7 (*0.4*)	1 (*1.0*)	1 (*0.2*)	0.678	
Heart disease	18 (*1.1*)	1 (*0.4*)	9 (*1.6*)	0.308	
Current diabetes treatment	5 (*0.3*)	1 (*0.4*)	5 (*0.9*)	0.232	
Current dyslipidemia treatment	7 (*0.4*)	1 (*0.4*)	5 (*0.9*)	0.448	
Kidney disease	25 (*1.7*)	3 (*1.2*)	11 (*2.1*)	0.69	
History of smoking-*n* (*%*)	302 (*19.1*)	26 (10.0)	78 (13.7)	< 0.001	< 0.001 for A *vs.* B, and 0.005 for A *vs.* C
Drinking habit of alcohol-*n* (*%*)	630 (*38.1*)	74 (26.8)	166 (28.2)	< 0.001	< 0.001 for A *vs.* B, and A *vs.* C
**Relocated after the disaster-*n* (*%*)**					
To other municipalities	1,066 (62.8)	176 (62.2)	356 (59.2)	0.297	
To other prefectures	303 (17.9)	54 (19.1)	95 (15.8)	0.4	
**Hematological characteristics**					
History diagnosed as anemia-*n* (*%*)	162 (*10.1*)	70 (26.3)	164 (28.8)	< 0.001	< 0.001 for A *vs.* B, and A *vs.* C
**Peripheral blood counts**					
Red blood cells (10^6^/μL)	4.58 (*0.30*)	4.39 (*0.38*)	4.46 (*0.35*)	< 0.001	< 0.001 for A *vs.* B, A *vs.* C, and 0.004 for B *vs*. C
Hemoglobin (g/dL)	13.7 (*0.8*)	12.1 (*1.3*)	12.2 (*1.4*)	< 0.001	< 0.001 for A *vs.* B and A *vs.* C
Hematocrit (%)	41.2 (*2.2*)	37.7 (*3.1*)	38.0 (*3.4*)	< 0.001	< 0.001 for A *vs.* B and A *vs.* C
**Red cell indices**					
MCV (fl)	90.1 (*4.0*)	86.3 (*7.6*)	85.5 (*7.4*)	< 0.001	< 0.001 for A *vs.* B, and A *vs.* C
MCV < 80-*n* (*%*)	21 (1.2)	49 (17.3)	129 (21.5)	< 0.001	< 0.001 for A *vs.* B, and A *vs.* C
MCV > 100-*n* (*%*)	18 (1.1)	2 (0.7)	2 (0.3)	0.239	
MCH	29.9 (*1.5*)	27.8 (*3.2*)	27.5 (*3.2*)	< 0.001	< 0.001 for A *vs.* B, and A *vs.* C
MCHC	33.1 (*0.9*)	32.2 (*1.5*)	32.1 (*1.5*)	< 0.001	< 0.001 for A *vs.* B, and A *vs.* C

No significant differences were detected among the three groups with respect to medical histories. However, the smallest proportions of smoking history and alcohol consumption were found in the anemia recovered group (10.0% and 26.8%, respectively; *p* < 0.001 between the anemia recovered and no anemia groups for both comparisons). Of note, no significant differences were detected in the moving status after the disaster among the three groups. Overall, approximately 60% of participants moved to other municipalities and less than 20% of those to the other prefectures.

A significantly higher proportion with a diagnostic history of anemia before the disaster was seen in the anemia recovered and non-recovered groups when compared to the no anemia group (26.3, 28.8, and 10.1%, respectively; *p* < 0.001 for either anemia recovered or non-recovered groups vs. no anemia group). Peripheral blood counts at baseline revealed significantly lower hemoglobin concentrations and smaller mean corpuscular volume in the recovered and non-recovered groups than in the no anemia group (12.1, 12.2, and 13.7 g/dL, and 86.3, 85.5, and 90.1 fL, respectively; *p* < 0.001 for both comparisons).

### Predictors of baseline characteristics for temporarily anemic status after the disaster in women

We explored the baseline characteristics to predict the temporarily anemic status where the anemia was observed in the early and/or middle phases and not in the late phase after the disaster. We selected age, BMI, systolic blood pressure, smoking history, drinking habit, and history of relocation for the candidates of explanatory variables. The body weight and waist circumstance were removed from further analysis due to multicollinearity with BMI. We categorized the BMI as less than 23 kg/m^2^ or larger ^35^. Disease histories were also removed because of their very small number, which causes inadequate statistical power.

The univariate logistic regression analysis revealed the BMI < 23 kg/m^2^, no history of smoking, and no habit of alcohol for statistically significant variables (odds ratio [*p* value]; 1.93 [< 0.001], 1.93 [0.002], and 1.50 [0.004], respectively) (Table 3). These three variables were further evaluated with a multivariate logistic regression model. The multivariate regression model exhibited BMI < 23 kg/m^2^, no history of smoking, and no habit of alcohol as independent predictors (adjusted odds ratio [95% confidence interval] and p-value; 1.98 [1.43–2.74] and *p* < 0.001, 1.85 [1.21–2.83] and *p* = 0.004, and 1.42 [1.07–1.90] and *p* = 0.015) (Hosmer–Lemeshow goodness-of-fit test: χ^2^ = 0.42, df = 4, *p* = 0.981).

**Table 3:** Results of the regression analysis to predict the temporarily anemic status after the disaster in women.

Variables	Temporarily anemic status		Univariate analysis		Multivariate analysis			
					The first step in the stepwise selection		Last step in the stepwise selection	
	Observed (*n* = 283)	Not observed (*n* = 2,298)	OR (95% CI)	*p* value	aOR (95%CI)	*p* value	aOR (95%CI)	*p* value
Age (years)	32 (5)	32 (5)	1.00 (0.97–1.02)	0.695	1.00 (0.97–1.03)	0.961	–	–
BMI < 23 kg/m^2^	231 (82%)	1,602 (70%)	1.93 (1.41–2.64)	< 0.001	1.85 (1.32–2.61)	< 0.001	1.98 (1.43–2.74)	< 0.001
Systolic blood pressure (mmHg)	110 (12)	111 (13)	0.99 (0.98–1.00)	0.06	0.99 (0.98–1.01)	0.27	–	–
No history of smoking	234 (90%)	1,769 (82%)	1.93 (1.27–2.94)	0.002	1.85 (1.21–2.83)	0.004	1.85 (1.21–2.83)	0.004
No habit of alcohol drinking	202 (73%)	1,445 (65%)	1.50 (1.14–1.99)	0.004	1.41 (1.06–1.89)	0.018	1.42 (1.07–1.90)	0.015
**Relocated after the disaster**								
No relocation	107 (38%)	876 (38%)	1 (ref)	0.751	1 (ref)	0.694	–	–
To other municipalities	122 (43%)	1,024 (45%)	0.98 (0.74–1.28)	0.859	1.00 (0.75–1.33)	0.997		
To other prefectures	54 (19%)	398 (17%)	1.11 (0.78–1.57)	0.554	1.17 (0.79–1.72)	0.433		

Data are shown as the average (standard deviation ) or numbers (percentage).

*BMI* body mass index, *95%CI* 95% confidence interval, *aOR* adjusted odds ratio, *OR* odds ratio.

## Discussion

This study shows the declining trend of the proportion of anemia in young women evacuated after the Great East Japan Earthquake. The findings presented here are important from a public health perspective since the anemia cause fatigue, weakness, and reduced exercise performance in the general population but also increases the risk of preterm birth and low birth weight infants if pregnant women develop anemia^36,37^. Researchers in the Centers for Disease Control and Prevention (CDC) reported that the prevalence of anemia had increased by 0.4% (from 0.7 to 1.1%) in pregnant women who gave birth after the Red River flood^9^; however, the study did not continuously monitor post-disaster anemia and did not provide sufficient information on this issue. Several other studies, including the nuclear accident in Chornobyl, have been published on post-disaster anemia, but all of them have similar limitations, which is the lack of longitudinal data^10–12^. The FHMS might have contributed to solving the limitations since FMHS is an annual survey of all Fukushima residents, lasting for more than a decade, from immediately after the disaster to the present. However, we should carefully interpret the results in this study since the participation rate of the FHMS has largely dropped down, as discussed later.

It is noteworthy that the proportion of anemia in women showed a declining trend after the disaster. There are several possible explanations for this trend in anemia proportion. First, the nutritional status, especially for iron intake, might contribute declining trend of anemia proportion. Iron deficiency is the most common cause of anemia, and high-heme food like animal meat and fish is a major source of iron intake in general^38^. Zhang et al. reported the significantly poor dietary intake of meat and fish among evacuees early after the Great East Japan Earthquake using the FHMS data^39^. The reduction in meat and fish intake would have led to a lower heme iron intake, which ultimately contributes to anemia. The social background of reduction in high heme-food intake includes preserved foods^23,40^. Curbs on fishing after the FDNPP accident might contribute to the poor intake of fish as fisheries were a major industry in the area surveyed in this study^41^. Additionally, residents in Fukushima developed a tendency to avoid potentially contaminated local foods such as meat, fish, mushrooms, and milk^24^. Overall, poor intake of high heme food early after the disaster might improve over time, which in turn led to a declining trend in the anemia proportion. Second, the post-disaster difficulties in accessing iron supplement medication and low-dose oral contraceptives might exist, although no study has been published on these concerns after the disaster, and FHMS did not collect such data. Third, pregnancy can influence anemic status. However, the number of births decreased sharply after the disaster in Fukushima^42^. Therefore, it is unlikely that the temporary increase in the proportion of anemia was due to the effect of pregnancy. Also, exposure to radioactive materials generally increases the prevalence of hematopoietic malignancies. However, no increase in the prevalence of malignant tumors has been observed to date^43^.

Furthermore, the amount of cesium-137 released from the Fukushima Daiichi Nuclear Power Plant was 0.38 times less than that of the Chornobyl disaster^44,45^, and strict radiation countermeasures have been continuously employed^46–48^. Finally, several infectious diseases, including parasites and human immunodeficiency virus, and some hematological diseases, such as thalassemia, also cause anemia. Again, however, their incidence in Japan is low, and there have been no reports indicating an increase for these in Fukushima since the disaster. Therefore, we conclude that the impact of these factors is negligible in the present study.

The multivariate regression analysis showed that not obese women without smoking and drinking habits are more likely to suffer from anemia in the early and/or middle phases after the disaster. The findings urged us to postulate that women evacuees with healthier habits before the disaster were at high risk of anemia. Our previous report showed a higher risk of anemia in young women with a lower BMI in the Asian cohort consisted with this study^6^. Caution for the interpretation of the analysis on the temporarily anemic status is an aging effect on anemia where the prevalence of anemia increases with age due to the increased incidence of hemorrhagic diseases, such as uterine fibroids^49–53^. We equally treated anemic status among early, middle, and late phases after the disaster for regression analysis; however, there might be a bias of time-dependent increase in the anemia proportion due to the aging effect.

While we believe that this study provides valuable information on disaster-related anemia, it has limitations. Notably, the number of study subjects decreased as the study progressed, dropping from 9,198 participants in 2011 to 1,241 in 2018. This creates a strong potential for selection bias in our results as those who continued to receive medical checkups, and those who stopped receiving them may exhibit markedly different backgrounds. In fact, we observed smaller MCV at baseline in the subjects without participation after 2012 when compared to those who participated once or more. Also, people who routinely undergo annual physical examinations are more likely to be concerned about their health and might have a lower frequency of anemia. We might have underestimated the proportion of anemia in the middle and late phases of our study. We are currently performing a further analysis to elucidate the association between participant characteristics and participation status. Second, it is difficult to follow up on those who evacuated outside of the Fukushima Prefecture, as the Prefecture government conducted the survey. There also exists the potential that those who evacuated outside of Fukushima Prefecture are more likely to fear radiation and suffer from various health problems, including psychological distress^54^, than those who relocated within Fukushima Prefecture. We cannot deny the possibility of underestimating the proportion of anemia. Third, we did not link the health checkup data with their pregnancy since the pregnant women have been surveyed by a different program in FHMS. Pregnancy is a potent variable that can develop anemia. We might fail to identify the influence of pregnancy on the anemia proportion in this study. Fourth, the questionnaire used in the FHMS did not reflect the lifestyle changes of young men and women. For instance, questions about dietary preferences relating to vegetarianism, supplement use, and low-dose oral contraceptives were lacking. Administering questionnaires addressing these factors would facilitate more detailed analyses. Finally, the lack of health data before the Great East Japan Earthquake made us difficult to evaluate the direct effect of the disaster on anemic status.

## Conclusion

This study revealed that the proportion of anemia among young women who participated in the comprehensive health checkup of FHMS declined for eight years after the great earthquake in 2011. Young women with low BMI and healthier habits of no smoking and drinking might risk temporarily anemic condition after the disaster. A major limitation of this study is the lowering of the participation rate of the FHMS. Further large-scale studies are needed to clarify these findings and to reduce the negative public health impact of anemia after the disaster.

## Supplementary Information

Supplementary Information.

## Data Availability

The datasets analysed during the current study are not publicly available due to the privacy policy of the Fukushima Health Management Survey belongs to the government of Fukushima Prefecture but are available from the corresponding author on reasonable request.
