# Supplementary material for: Changes in the proportion of anemia among young women after the Great East Japan Earthquake: the Fukushima health management survey
Source: Sci Rep. 2022 Jun 25;12:10805. doi: 10.1038/s41598-022-14992-3 (PMC9233683; doi:10.1038/s41598-022-14992-3)
Supplement: Supplementary file 1 — Supplementary Information. [file 41598_2022_14992_MOESM1_ESM.pdf]

Supporting information

S1 Fig. Longitudinal follow-up of study participants.

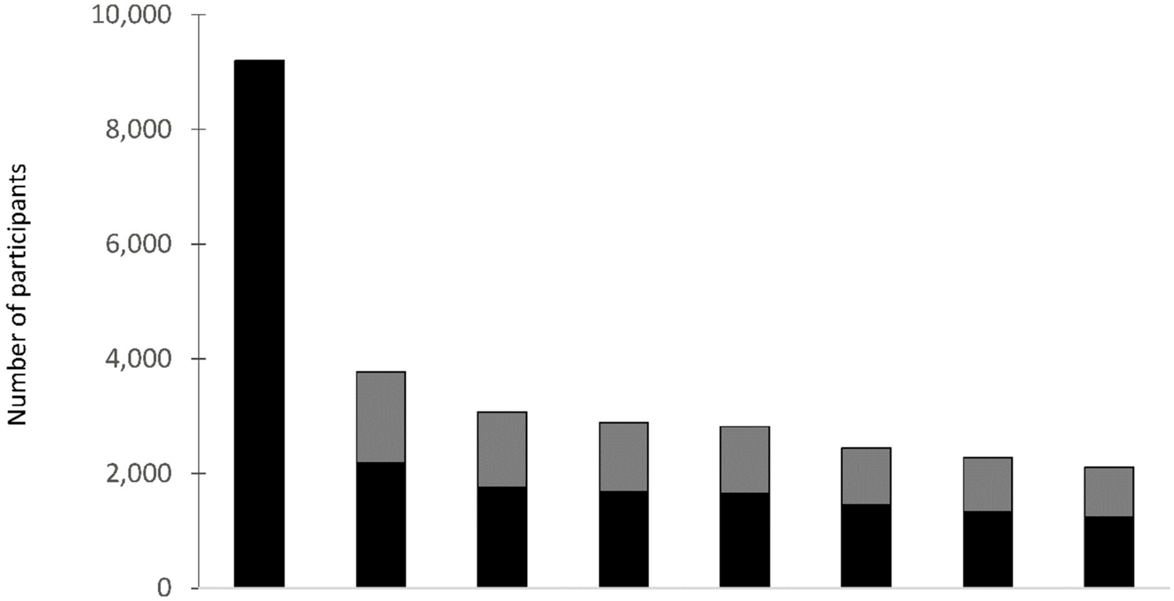

Year	2011	2012	2013	2014	2015	2016	2017	2018
Total number of participants by year	9,198	3,774	3,077	2,892	2,820	2,444	2,280	2,116
Those who participated in 2011 health check	—	2,192 (*58*)	1,761 (*57*)	1,685 (*58*)	1,653 (*59*)	1,458 (*60*)	1,341 (*59*)	1,241 (*59*)

The number of study participants is shown by survey year. Black shading indicates individuals who participated in the 2011 health check, and the grey shading those continuing to receive health checks between 2012 and 2018.

S2 Fig. Proportion of anemia by age groups in young women participated in the Fukushima Health Management Survey.

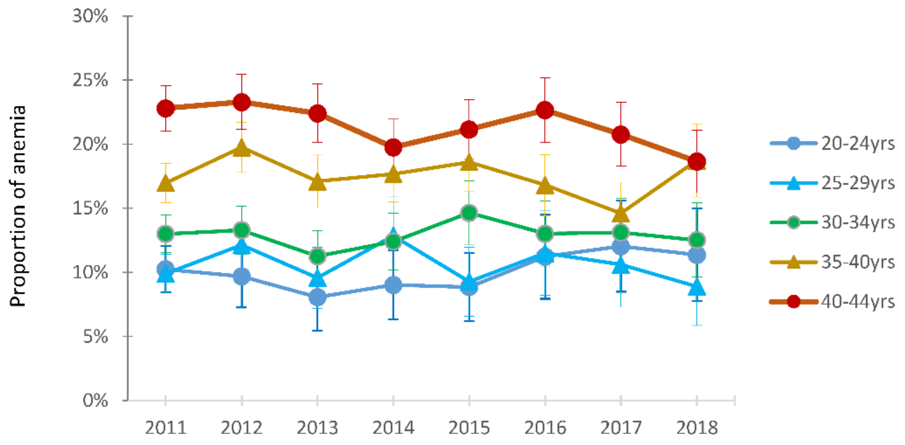

The proportion of anemia among the women participants is shown by age group. The bars indicate 95% confidence intervals.

S1 Table. The annual participation rate of the comprehensive health checkup program in the Fukushima Health Management **Survey.**

Fiscal year	2011	2012	2013	2014	2015	2016	2017	2018
All age								
Number of target population	210,189	211,987	213,444	214,211	215,315	215,701	215,296	214,718
Number of participants	74,333	58,789	53,288	51,090	48,814	46,403	44,770	43,294
Participation rate	35.4%	27.7%	25.0%	23.9%	22.7%	21.5%	20.8%	20.2%
Individuals aged 16 years or older								
Number of target population	182,370	184,910	186,970	188,328	190,019	191,101	191,636	191,974
Number of participants	56,399	47,009	43,040	41,874	41,211	39,990	39,367	38,815
Participation rate	30.9%	25.4%	23.0%	22.2%	21.7%	20.9%	20.5%	20.2%
Individuals aged 15 years or younger								
Number of target population	27,819	27,077	26,474	25,883	25,296	24,600	23,660	22,744
Number of participants	17,934	11,780	10,248	9,216	7,603	6,413	5,403	4,479
Participation rate	64.5%	43.5%	38.7%	35.6%	30.1%	26.1%	22.8%	19.7%

The annual numbers of the target population and participants of the comprehensive health checkup program are shown. The participation rate (percentage) is also displayed. Data were obtained from the website of the Fukushima Prefecture;
https://www.pref.fukushima.lg.jp/uploaded/attachment/369432.pdf
(Accessed on May 4, 2022)

S2 Table. Estimation of the population eligible for the comprehensive health checkup program of the Fukushima Health Man**agement Survey in March 2011.**

	Age group					Total
	20–24 yrs	25–29 yrs	30–34 yrs	35–39 yrs	40–44 yrs	
Total	3,797	4,736	5,336	5,740	5,449	25,058
Municipal district						
Tamura City	844	904	890	910	1,138	4,686
Minami-Soma City	1,199	1,574	2,033	2,257	1,889	8,952
Kawamata Town	255	323	332	395	355	1,660
Hirono Town	105	135	130	125	138	633
Naraha Town	165	162	210	199	190	926
Tomioka Town	308	414	447	521	456	2,146
Kawauchi Village	38	42	63	48	62	253
Ohkuma Town	259	329	370	383	310	1,651
Futaba Town	118	151	185	204	180	838
Namie Town	382	542	514	519	552	2,509
Katsurao Village	25	21	36	23	31	136
IIdate Village	85	124	110	135	129	583
Part of Date City*	14	15	16	21	19	85

Data were obtained from the Fukushima Prefecture Population Survey conducted in March 2011 (https://www.pref.fukushima.lg.jp/sec/11045b/15859.html).

*A part of Date City was designated as an evacuation zone, where 413 people lived according to the 81st Disaster Control Meeting of the Date City on July 1, 2011. (The meeting minutes are available at https://www.city.fukushima-date.lg.jp/uploaded/attachment/1664.pdf). We estimated the population in the evacuation zone in Date City using the population of the same city classified by age group.

S3 Table. Comparison of baseline characteristics in 2011 grouped by participation status after 2012 among young women in the Fukushima Health Management Survey.

Baseline characteristics in 2011	Participation in the FHMS after 2012		*p* value
	Participated once or more (*n* = 5,577)	No participation (*n* = 3,621)	
Age — years	33.5 (*6.2*)	34.0 (*7.5*)	0.002
Body weight (kg)	55.1 (*10.6*)	56.2 (*11.4*)	<0.001
Body mass index (kg/m^2)	22.1 (*4.1*)	22.5 (*4.3*)	<0.001
Medical history — *n* (*%*)			
Current hypertension	81 (*1.5*)	64 (*1.8*)	0.253
Cerebrovascular disease	12 (*0.2*)	8 (*0.2*)	0.968
Heart disease	66 (*1.2*)	30 (0.8)	0.093
Current diabetes treatment	33 (*0.6*)	22 (*0.6*)	0.947
Current treatment of dyslipidemia	34 (*0.6*)	25 (*0.7*)	0.661
Kidney disease	110 (*2.0*)	60 (*1.7*)	0.273
History of smoking — *n* (*%*)	1,019 (*18.8*)	778 (*21.9*)	<0.001
Drinking habit of alcohol — *n* (*%*)	472 (*8.5*)	342 (*9.4*)	0.105
Hematological characteristics			
History diagnosed as anemia — *n* (*%*)	943 (*17.5*)	667 (*18.9*)	0.094
Peripheral blood counts			
Red blood cells (10^6/μL)	4.54 (*0.33*)	4.54 (*0.33*)	0.242
Hemoglobin (g/dL)	13.1 (*1.3*)	13.1 (*1.4*)	0.484
Hematocrit (%)	40.0 (*3.2*)	39.9 (*3.4*)	0.334
Red cell indices			
MCV (fl)	88.2 (*6.1*)	87.9 (*6.6*)	0.019
MCH	29.0 (*2.6*)	28.9 (*2.9*)	0.079
MCHC	32.8 (*1.3*)	32.8 (*1.4*)	0.628

The average (standard deviation) or numbers (percentages) are shown. Abbreviations: mean corpuscular volume; MCV, mean corpuscular hemoglobin; MCH, and mean corpuscular hemoglobin concentration; MCHC. The statistical evaluation was performed with Student-t or Pearson Chi-square tests on the continuous or categorical variables.

S4 Table. The number of participants with missing data from the 2011 comprehensive health check.

	Women (*n* = 9,198)
Age	0 (*0.0*)
Body weight	0 (*0.0*)
Body mass index	0 (*0.0*)
Medical history	
Current hypertension treatment	260 (*2.8*)
Cerebrovascular disease	266 (*2.9*)
Heart disease	269 (*2.9*)
Current diabetes treatment	236 (*2.6*)
Current treatment of dyslipidemia	252 (*2.7*)
Kidney disease	261 (*2.8*)
History of smoking	213 (*2.3*)
Daily alcohol consumption	204 (*2.2*)
Hematological characteristics	
History diagnosed as anemia	270 (2.9)
Peripheral blood count	0 (*0.0*)

Number (percentage) of participants with missing values is shown.

S5 Table. A comparison of women participant characteristics at baseline classified by disease period.

Group	A	B	C	D	E	F	G	H	*p*-value
	No anemia	Anemia groups							
		Anemia recovered group			Anemia non-recovered group				
		Early phase only	Middle phase only	Early and Middle phases	Late phase only	Early and Late phases	Middle and Late phases	Persistent anemia	
	(*n* =1,697)	(*n* =107)	(*n* =110)	(*n* =66)	(*n* =186)	(*n* =52)	(*n* =133)	(*n* =230)	
Age at the disaster in 2011	30.7 (*5.0*)	30.6 (*4.9*)	31.0 (*5.2*)	31.5 (*5.6*)	31.0 (*5.1*)	31.2 (*5.6*)	31.7 (*4.8*)	33.1 (*4.5*)	A:H***, B:H***, C:H*, E:H***
Body weight (kg)	55.5 (*11.5*)	53.8 (*9.0*)	53.0 (7.4)	52.9 (9.6)	56.0 (11.5)	55.5 (10.3)	54.9 (12.3)	53.9 (9.8)	A:C*
Body mass index (kg/m^2)	22.2 (*4.4*)	21.5 (*3.8*)	21.1 (2.7)	20.9 (3.5)	22.5 (4.5)	22.4 (4.0)	21.8 (4.7)	21.6 (3.7)	A:C**, C:E*
Waist (cm)	77.1 (10.4)	76.3 (12.0)	74.2 (6.7)	71.2 (7.6)	78.1 (8.8)	74.8 (11.1)	78.5 (12.3)	74.2 (8.4)	A:D*, D:D*
Systolic blood pressure (mmHg)	111 (12)	108 (11)	111 (13)	111 (11)	112 (13)	113 (13)	112 (14)	113 (13)	B:H**
Diastolic blood pressure (mmHg)	68 (10)	64 (9)	69 (10)	66 (9)	68 (10)	67 (12)	68 (11)	68 (10)	A:B**, B:C*, B:E**, B:G*, B:H*,
Medical history — *n* (*%*)									
Medication for hypertension	15 (*0.9*)	2 (*1.9*)	0 (0.0)	1 (1.6)	1 (0.6)	0 (0.0)	3 (2.3)	5 (2.2)	*n.s.*
Cerebrovascular disease	7 (*0.4*)	1 (*1.0*)	0 (0.0)	0 (0.0)	0 (0.0)	0 (0.0)	0 (0.0)	1 (0.5)	*n.s.*
Heart disease	18 (1.1)	0 (*0.0*)	0 (*0.0*)	1 (1.6)	2 (1.1)	2 (4.1)	1 (0.8)	4 (1.8)	*n.s.*

Current diabetes treatment	5 (*0.3*)	0 (*0.0*)	0 (*0.0*)	1 (1.8)	1 (0.6)	0 (*0.0*)	0 (*0.0*)	4 (1.8)	*n.s.*
Current dyslipidemia treatment	7 (*0.4*)	0 (*0.0*)	0 (*0.0*)	1 (1.8)	2 (1.2)	0 (*0.0*)	2 (1.6)	1 (0.5)	*n.s.*
Kidney disease	25 (*1.7*)	3 (*3.2*)	0 (*0.0*)	0 (*0.0*)	1 (0.6)	4 (8.9)	1 (0.9)	5 (2.4)	*n.s.*
History of smoking — *n* (*%*)	302 (*19.1*)	9 (*8.8*)	11 (10.9)	6 (10.5)	27 (15.3)	5 (10.4)	18 (14.0)	28 (12.9)	*n.s.*
Alcohol consumption frequency— *n* (*%*)	630 (*38.1*)	21 (*20.2*)	32 (29.6)	21 (32.8)	61 (33.9)	18 (35.3)	30 (23.1)	57 (25.1)	A:B**, A:G*, A:H**
Relocated after the disaster — *n* (*%*)									
Moved to other municipalities	1,066 (62.8)	67 (62.6)	68 (61.8)	41 (62.1)	107 (57.5)	33 (63.5)	85 (63.9)	131 (57.0)	*n.s.*
Moved to other prefectures	303 (17.9)	13 (12.1)	22 (20.0)	19 (28.8)	27 (14.5)	10 (19.2)	18 (13.5)	40 (17.4)	*n.s.*
Hematological characteristics									
History of anemia diagnosis — *n* (*%*)	162 (*10.1*)	25 (*25.3*)	23 (22.1)	22 (34.9)	26 (14.7)	19 (38.8)	36 (29.3)	83 (37.6)	A:B**, A:C*, A:E***, A:F***, A:G***, A:H***, D:E*, E:F*, E:H***
Peripheral blood counts									
Red blood cells (10^6/μL)	4.58 (*0.30*)	4.28 (*0.42*)	4.48 (0.29)	4.42 (0.38)	4.53 (0.32)	4.33 (0.45)	4.51 (0.30)	4.41 (0.37)	A:B***, A:C*, A:D*, A:F**, A:H***, B:C**, B:E***, B:G***, E:H*,
Hemoglobin (g/dL)	13.7 (*0.8*)	11.8 (*1.1*)	13.1 (0.6)	11.1 (1.3)	13.3 (0.7)	11.5 (1.3)	13.0 (0.7)	11.1 (1.2)	A:B***, A:C***, A:D***, A:E***, A:F***, A:G***, A:H***, B:C***, B:E***, B:E**, B:G***, B:H***, C:E**, C:F***, C:H**, D:E***, E:F***, E:G**, E:H***, D:G**, F:G***, G:H**
Hematocrit (%)	41.2 (*2.2*)	36.7 (*2.8*)	40.0 (1.8)	35.7 (3.1)	40.4 (2.2)	36.1 (3.1)	39.8 (2.0)	35.6 (2.9)	A:B**, A:C***, A:D***, A:E**, A:F***, A:G***, A:H***, B:C***, B:E***, B:G***, B:H*, C:E***,

									C:F***, C:H**, D:E***, E:F***, E:H***, D:G***, F:G***, G:H**
Red cell indices									
MCV (fl)	90.1 (*4.0*)	86.2 (*6.8*)	89.5 (5.1)	81.3 (9.6)	89.4 (4.1)	84.1 (9.2)	88.5 (4.9)	80.9 (7.7)	A:B***, A:E***, A:F***, A:G**, A:H***, B:C**, B:D***, B:E*, B:H***, C:E***, C:F**, C:H***, D:E***, E:F**, E:H**, D:G***, G:H**
MCV <80 — *n* (*%*)	21 (1.2)	16 (15.0)	4 (3.6)	29 (43.9)	5 (2.7)	14 (26.9)	9 (6.8)	101 (43.9)	A:B***, A:E***, A:F***, A:G**, A:H***, B:C*, B:D**, B:E**, B:H***, C:E***, C:F**, C:H***, D:E***, E:F***, E:H***, D:G***, F:G**, G:H***
MCV >100 — *n* (*%*)	18 (1.1)	1 (0.9)	1 (0.9)	0 (0.0)	1 (0.5)	0 (0.0)	0 (0.0)	1 (0.4)	*n.s.*
MCH	29.9 (*1.5*)	27.8 (*2.9*)	29.4 (1.9)	25.3 (3.9)	29.4 (1.5)	26.9 (4.1)	28.8 (1.8)	25.3 (3.3)	A:B***, A:D***, A:E***, A:F***, A:G***, A:H***, B:C***, B:D***, B:E***, B:H***, C:E***, C:F**, C:H**, D:E***, E:F**, E:H***, D:G***, G:H**
MCHC	33.1 (*0.9*)	32.3 (*1.3*)	32.8 (1.1)	31.0 (1.7)	32.9 (0.9)	31.8 (1.8)	32.6 (0.9)	31.2 (1.5)	A:B***, A:C*, A:D***, A:E***, A:F***, A:G***, A:H**, B:C*, B:D***, B:E***, B:H***, C:E***, C:F*, C:H**, D:E***, E:F**, E:H**, D:G***, G:H**

The numerical and categorical data were statistically evaluated with a one-way analysis of variance (ANOVA) followed by Dunnett T3 test and Fisher's exact test with Holm correction, respectively. *p <0.05, **p <0.01, ***p <0.001.
